# Rationale and design of study of dapagliflozin versus sitagliptin treatment efficacy on prevention of cardiovascular risk factors in type 2 diabetes patients: the DIVERSITY-CVR study

**DOI:** 10.1186/s12933-018-0730-z

**Published:** 2018-06-12

**Authors:** Fumika Shigiyama, Naoki Kumashiro, Ayako Fuchigami, Takahisa Hirose

**Affiliations:** Division of Diabetes, Metabolism, and Endocrinology, Department of Medicine, Toho University Graduate School of Medicine, 6-11-1 Omori-Nishi, Ota-ku, Tokyo, 143-8541 Japan

**Keywords:** Dapagliflozin, Sitagliptin, Cardiovascular diseases, Type 2 diabetes

## Abstract

**Background:**

Recent studies reported that sodium glucose cotransporter 2 (SGLT2) inhibitors reduced the cardiovascular morbidity and mortality in patients with type 2 diabetes mellitus (T2DM) compared to placebo in contrast to no reduction with dipeptidyl peptidase 4 (DPP4) inhibitors. However, there are no comparative studies on the effects of SGLT2 inhibitors and DPP4 inhibitors on HbA1c, body weight and hypoglycemia as risk factors of cardiovascular diseases. The aim of the present ongoing study is to compare the effects of dapagliflozin, a SGLT2 inhibitor, with those of sitagliptin, a DPP4 inhibitor, on cardiovascular risk factors in T2DM patients with inadequate glycemic control.

**Methods:**

The study of dapagliflozin versus sitagliptin treatment efficacy on prevention of cardiovascular risk factors in T2DM patients (DIVERSITY-CVR study) is a prospective, randomized, open-label, blinded-endpoint, parallel-group, comparative study. A total of 340 T2DM patients treated with metformin alone or with no glucose-lowering agents (hemoglobin A1c ≥ 7.0 and < 10.0%) will be randomized into the dapagliflozin group (5–10 mg/day, n = 170) and the sitagliptin group (50–100 mg/day, n = 170), and treated for 24 weeks. The primary endpoint is the rate of achieving a composite endpoint of the following three items at 24th week; (1) HbA1c < 7.0%; (2) body weight loss of ≥ 3.0% from baseline; (3) avoidance of hypoglycemia. Hypoglycemia will be monitored using the flash glucose monitoring system. The secondary outcomes include each component of the primary endpoint, plus indices of lipid metabolism, and evaluations related to safety.

**Conclusions:**

There is lack of solid information on differences in the therapeutic effects of SGLT2 inhibitors and DPP4 inhibitors on multiple risk factors for cardiovascular diseases. It is anticipated that the results of the DIVERSITY-CVR study provides useful clinical data on the management of patients with T2DM, including reducing the risk of CVD. The results of this study will become available in 2019.

*Trial registration* University Hospital Medical Information Network Clinical Trial Registry (UMIN000028014). Registered 30 June 2017

**Electronic supplementary material:**

The online version of this article (10.1186/s12933-018-0730-z) contains supplementary material, which is available to authorized users.

## Background

Type 2 diabetes mellitus (T2DM) is a high risk factor for cardiovascular diseases (CVD) [1–4], and a major cause of mortality worldwide [5]. Hyperglycemia can cause abnormal tissue function and lead to endothelial dysfunction, plaque formation, structural alteration of arterial tissue and atherosclerosis [6–9]. Previous studies reported that 1% reduction in hemoglobin A1c (HbA1c) concentration was associated with significant reductions in diabetes-related deaths (21%) and myocardial infarction (14%) [10]. However, severe hypoglycemia in patients with T2DM is also known to have negative effects on the incidence of CVD and mortality risk [11–13]. Previous meta-analysis study of 903,510 patients with T2DM reported that the odds ratio for CVD due to severe hypoglycemia was 2.05-fold [13]. Obesity is another risk factor for CVD [14–16], and is a well-established risk factor for T2DM [17]. Obesity causes ectopic fat accumulation and this leads to insulin resistance [18]. Insulin resistance is one of the major pathogenic factor of T2DM and progression of CVD [19]. Thus, the management of T2DM patients should focus on lowering blood glucose and body weight without hypoglycemia.

Regarding the choice of treatment, various oral glucose-lowering agents of different mechanisms are available. Sodium glucose cotransporter 2 (SGLT2) inhibitors, can prevent elevation of blood glucose levels by suppression of the reuptake of sodium and glucose from primitive urine [20]. In addition to glucose-lowering effects, empagliflozin and canagliflozin, two SGLT2 inhibitors, have been demonstrated on their protective role on cardiovascular events among patients with T2DM [21–23]. In addition, Dapagliflozin Effect on CardiovascuLAR Events [DECLARE TIMI-58]; NCT01730534, which examines the effect of dapagliflozin, another SGLT2 inhibitor used in this study, on cardiovascular outcome, has been ongoing and the result will be available soon [24]. In this regard, we have demonstrated that dapagliflozin can correct endothelial dysfunction compared to metformin in T2DM with moderate hyperglycemia [25]. In contrast to SGLT2 inhibitors, few randomized clinical trials have shown the beneficial effects of dipeptidyl peptidase 4 (DPP4) inhibitors on cardiovascular events in patients with T2DM compared to placebo control [23, 26–28]. However, sitagliptin, a DPP4 inhibitor, was shown to lower the rate of newly diagnosed CVD in T2DM [29] and we also demonstrated that linagliptin, another DPP4 inhibitor, improved endothelial function, as assessed by flow-mediated dilation in patients with T2DM [30].

To date, there are few prospective randomized clinical trials that have compared the efficacy of SGLT2 inhibitors and DPP4 inhibitors regarding the prevention of CVD risks, such as hyperglycemia, body weight gain and severe hypoglycemia. Therefore, we planned and partly executed this clinical trial aimed to compare the therapeutic benefits of dapagliflozin and sitagliptin on CVD risks, with special focus on HbA1c, body weight and hypoglycemia. Hypoglycemia will be monitored by the flash glucose monitoring system (FGM) (FreeStyle Libre Pro; Abbott Diabetes Care, Tokyo, Japan), which continuously records subcutaneous interstitial glucose concentrations for 14 days without the need for re-calibration and has been used for the assessment of hypoglycemia [31]. The comparative study of treatment efficacy of dapagliflozin versus sitagliptin in reducing cardiovascular risk factors in T2DM patients: DIVERSITY-CVR study will provide better understanding of the effects of dapagliflozin and sitagliptin to establish an effective treatment strategy for T2DM and prevention of CVD.

## Methods

### Study design

The DIVERSITY-CVR study is an ongoing, prospective, randomized open-label, blinded-endpoint study, registered on the University Hospital Medical Information Network Clinical Trial Registry (UMIN000028014), a non-profit organization in Japan that meets the requirements of the International Committee of Medical Journal Editors (ICMJE). This study was approved by the Medical Ethics Committee of Toho University (approval #M17024). The study will be conducted according to the Declaration of Helsinki and current legal regulations in Japan. To avoid bias regarding the processes of enrollment, randomization, data collection and management are conducted by a third party.

### Study population

In this study, the target number of patients required for registration was set at 340 Japanese patients with T2DM who regularly visited the Outpatient Clinics of 62 institutions across Japan (listed in the Additional file 1). Recruitment for the study began in July 2017 and will end in June 2018. At the time of writing this report, a total of three subjects have completed the 24-week study, 178 subjects have been recruited and started the study, and 132 subjects are to be recruited during the remaining part of the study. The inclusion criteria were set as follows: (1) T2DM patients who have not used any glucose-lowering agents within 8 weeks before consenting, or those who have only used metformin (250–2250 mg/day), in addition to diet and exercise; (2) HbA1c (NGSP values) level of 7.1% or higher but no more than 10.0%; (3) males or females aged 20–80 years; (4) patients with body mass index (BMI) of 23 kg/m^2^ or higher; (5) patients who can be monitored closely for medication compliance; (6) patients who provide written informed consent. The following exclusion criteria are also used: (1) patients with type 1 diabetes or secondary diabetes; (2) patients with medical history of diabetic ketoacidosis; (3) patients with medical history of myocardial infarction, cerebral infarction, or stroke within 12 weeks before consent to the study; (4) patients with severe liver disease; (5) patients with renal disease [serum creatinine 1.3 mg/dL or higher, or estimated glomerular filtration rate (eGFR) less than 45 mL/min/1.73 m^2^]; (6) patients with unstable hypertension or dyslipidemia within 12 weeks before consent to the study; (7) pregnant or breastfeeding patients, or those planning to become pregnant during the study; (8) dehydrated patients [test results show abnormality in hematocrit or blood urea nitrogen (BUN) or complaint of dehydration].

### Randomization and study intervention

**Fig. 1:**
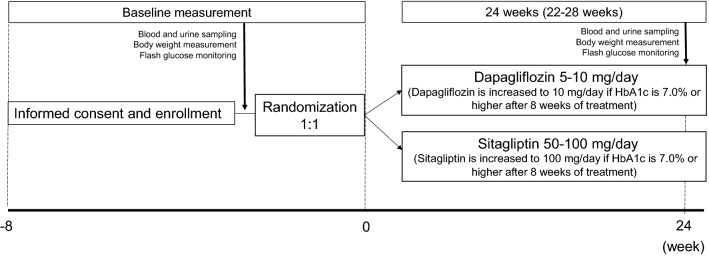
Patient recruitment process

Enrollment, randomization and follow-up schedule are outlined in Fig. 1. After consent and enrollment, the eligible subjects are randomly assigned at equal numbers into the dapagliflozin group (dapagliflozin 5–10 mg/day), and sitagliptin group (sitagliptin 50–100 mg/day). The randomization is conducted by a computer-based dynamic allocation method using HbA1c and BMI values collected at consent as the background factor for allocation. After enrolment, patients are asked to refrain from changing the dose of concomitant drugs or use additional medications during the 24-week study, such as other glucose-lowering agents, anti-hypertensive agents, lipid-lowering agents or antiplatelet agents. During the 8-week screening period, all baseline measurements of blood and urine samples and FreeStyle Libre Pro (Abbott Diabetes Care, Tokyo) (measurements of more than 5 days) are performed within 8 weeks after obtaining patients’ consent and before the allocation and administration of the study drug. After baseline data collection, patients of the dapagliflozin group are administered dapagliflozin 5 mg/day, which will be increased to 10 mg/day if HbA1c was 7.0% or higher after the 8 weeks. On the other hand, patients of the sitagliptin group are treated with sitagliptin 50 mg/day, which will be increased to 100 mg/day from 8 weeks if necessary to achieve the target HbA1c of < 7.0%. The treatment intervention start date is set as the observation start date. The assigned treatment is continued for 24 weeks (duration of the study).

### Study outcomes

The primary study endpoint is the rate of achieving a composite endpoint of the following three items from baseline to the 24th week; (1) HbA1c below 7.0%; (2) body weight loss of 3% relative to baseline; (3) avoidance of hypoglycemia [< 3.0 mmol/L (< 54 mg/dL)]. The secondary endpoints include the following items at 24 weeks: (1) rate of achieving HbA1c at < 7.0%; (2) rate of achieving body weight loss of ≥ 3% relative to baseline; (3) rate of avoiding hypoglycemia; (4) changes in HbA1c, fasting plasma glucose (FPG), body weight and BMI relative to baseline; (5) changes in lipid metabolism indices relative to baseline: triglyceride, total cholesterol, high-density lipoprotein (HDL) cholesterol and low-density lipoprotein (LDL) cholesterol; (6) changes in the levels of blood insulin, serum uric acid, BUN, serum creatinine, eGFR, aspartate aminotransferase (AST), alanine aminotransferase (ALT), serum sodium (Na), serum potassium (K), serum chloride (Cl) and blood cell count; (7) circadian change in glucose measured by FGM; (8) day-to-day variability in glucose by FGM; (9) frequency and duration of hypoglycemia; (10) medication adherence rate.

### Observation items and schedule

Clinical and biochemical data are collected at baseline and after the 24-week treatment period. Body weight is measured at hospital/clinic. All subjects wear the same disposable examination robe during body weight measurement. All blood samples for analyses are obtained after overnight fasting. The following items are measured using blood/urine samples; HbA1c, FPG, serum insulin, triglyceride, total cholesterol, HDL cholesterol, uric acid, BUN, creatinine, eGFR, AST, ALT, Na, K, Cl and blood cell count. LDL cholesterol is computed from the obtained data. In addition, to monitor the incidence of hypoglycemia, all subjects will wear the FGM (Freestyle Libre Pro; Abbott Diabetes Care, Tokyo) for 14 days at baseline and 24-week. In brief, the Freestyle Libre Pro device is attached on the upper arm of the subject, then glucose data are stored on the sensor every 15 min. Both the subjects and investigators are blinded to the glucose data during the study. After the 14-day glucose measurement, the subjects take off the sensor by themselves and send it to the data management third-party who is also blinded to the clinical information. The data management third-party downloads the glucose data from the sensor to the device software (Abbott Diabetes Care Inc.). All subjects are asked to record their daily intake of their medication using handy medication diary. Physicians check the record at subject’s every visit, and this record will continue through the study period and finally medication rate will be calculated using this record.

### Safety and evaluation of adverse events

During the course of the study, the investigators constantly monitor any adverse events (AEs) through regular medical checkups. Once an AE occurs, regardless of the presence or absence of a causal relationship with the study drug, the investigator reports the details immediately to the respective institution, the principal investigator and the administration office. All related AEs, not only side effects to the drug but also abnormal values from the clinical tests, are to be reported and documented.

### Sample size estimation

To our knowledge, there is no study that evaluated SGLT2 inhibitor on a composite endpoint (HbA1c below 7.0%, body weight loss of 3%, avoidance of hypoglycemia) as the primary outcome. Our previous study; the Relief study [30] and DEFENCE study [25], were used to select of rates of the above achievements for each group, and determine the required sample size for this study. In the Relief study [30], we referenced that 10.0% of patients that used a DPP-4 inhibitor, had HbA1c of 7.1% or higher and BMI of 23 kg/m^2^ or higher at baseline, could achieve the composite endpoint at 16 weeks. In the DEFENCE study [25], the proportion of subjects who used SGLT2 inhibitors and achieved the composite endpoint was 40.0% at 16 weeks. We estimated the achievement rates to be 25 and 40% for our sitagliptin and dapagliflozin groups, respectively. Based on these assumptions, the estimated sample size required to detect a significant difference in Chi square test between the two groups under the conditions of significance level of 5% on both sides and a power of 80%, was 152 cases per group. Assuming a dropout rate of 10%, the target number of enrolled patients was set at 170 cases per group for a total of 340 cases for the two groups.

### Statistical analysis

Analyses for the primary and secondary endpoints will be primarily performed on the full analysis set (FAS). FAS includes research subjects who are enrolled in this study and assigned to a study treatment, however, subjects without data for the primary endpoint or subjects with a significant study protocol violation are excluded. Safety analysis with adverse events is performed on the treated set. Summary statistics are calculated for continuous variables. For comparisons of the two groups, the Chi square test or Fisher’s exact test is used for nominal variables, and two sample *t* test or Wilcoxon ranked-sum test is used for continuous variables. For analysis of the primary endpoint, i.e., the proportion of participants who will be able to achieve all of the following: (1) HbA1c below 7.0% at 24th week, (2) body weight loss of ≥ 3% from baseline at 24th week, (3) no hypoglycemia [< 3.0 mmol/L (< 54 mg/dL)] throughout the study, comparison between the groups is performed using the Chi square test. For the secondary endpoint, the measured values and the extent of changes or percent changes, summary statistics are used on one-sample *t* test for comparisons within each group, and on two-sample *t* test for comparisons between groups. If the data deviate greatly from normal distribution, the Wilcoxon signed-rank test is used within each group, and Wilcoxon rank sum test is performed for comparisons between groups. All statistical analyses are to be performed independently by the administrative office of the DIVERSITY-CVR study using SAS software version 9.4 (SAS Institute, Cary, NC).

### Human rights and ethical principles of study subjects

All investigators involved in this study comply with the “World Medical Association Declaration of Helsinki” (2013 revision), and “Ethical Guidelines for Medical and Health Research Involving Human Subjects” (December 22, 2014, Ministry of Education, Culture, Sports, Science and Technology/Ministry of Health, Labor and Welfare), and other bylaws and regulations.

## Discussion

The DIVERSITY-CVR study is designed to compare the preventive effects of dapagliflozin and sitagliptin on CVD in T2DM patients. The rate of achieving a composite endpoint of the following three items at 24 weeks will be assessed as the primary endpoint; (1) HbA1c below 7.0%; (2) body weight loss of 3% from baseline; (3) avoidance of hypoglycemia [< 3.0 mmol/L (< 54 mg/dL)].

Several large prospective randomized trials on the cardiovascular outcomes have been performed but only few showed a significant effect for strict glycemic control on reduction of cardiovascular mortality, with the exception of the UK Prospective Diabetes Study (UKPDS) 34 on newly diagnosed obese T2DM patients [32–35]. Sub-analysis of the Action to Control Cardiovascular Risk in Diabetes (ACCORD) study showed that participants who had experienced symptomatic severe hypoglycemia were at greater risk of death than those who had experienced no hypoglycemia [36]. Another meta-analysis reported that strict blood glucose control was associated with adverse events of 2.5 kg weight gain and nearly doubled severe hypoglycemic episodes compared with standard treatment [37]. Taken together, these data suggest that glycemic control with severe hypoglycemic episodes and/or weight gain may lead to increased risk of CVD, and thus, we focused on the achievement of glycemic control without hypoglycemia and weight gain.

SGLT2 inhibitors are relatively new oral glucose-lowering agents and have attracted attention due to the recently reported improved cardiovascular outcomes in the large randomized controlled trials [21, 22]. In contrast, DPP4 inhibitors were reported neutral on cardiovascular outcomes in similarly large randomized controlled trials [26–28]. However, the mechanisms of improved cardiovascular outcomes by SGLT2 inhibitors are unclear, and the reason of difference on cardiovascular outcomes between SGLT2 inhibitors and DPP4 inhibitors is not well-addressed. Although we previously examined the effects of SGLT2 and DPP4 inhibition on endothelial function using flow-mediated dilation in T2DM patients treated with 750 mg metformin [25, 30], we could not compare the effects of SGLT2 and DPP4 inhibition directly and both agents tended to increase the flow-mediated dilation.

The present study compares the effects of dapagliflozin and sitagliptin on cardiovascular risk factors, focusing on achievement of HbA1c below 7.0%, 3% loss of body weight, and avoidance of hypoglycemia. Composite endpoints are being used frequently as outcomes, even as primary endpoint [38], for clinical trials in T2DM [39–41]. In addition, they have been adopted in a wide variety of clinical area especially CVD [39, 42, 43]. Unnikrishnan et al. [40] reviewed that composite endpoints have been preferred to assess the clinical benefit of intervention avoiding misinterpretation associated with competing risks factor bias and challenge of using a single outcome to validate the study objective in trials on patients with diabetes. The achievement of HbA1c below 7.0% is set as one of the components of the primary endpoint based on the following studies. The Diabetes Control and Complications trial study in type 1 diabetic patients showed significant reduction of microvascular complications in the intensive treatment group with HbA1c controlled to 7.1% [44]. The Kumamoto study also reported that prevention of the onset and progression of diabetic microangiopathy can be achieved by keeping HbA1c at < 6.9%, fasting blood glucose concentration at < 110 mg/dL and 2-h post-prandial blood glucose concentration at < 180 mg/dL [45]. In “Standards of Medical Care in Diabetes” of the American Diabetes Association, the target HbA1c for T2DM is set at around 7.0% [46]. Thus, the previous large clinical trials also evaluated the rate of achievement of HbA1c below 7.0% [27, 47, 48].

Another component of the primary endpoint is 3% loss in body weight. The Diabetes Prevention Program study demonstrated that body weight decrease of 1 kg contributed to 16% reduction in risk of diabetes [49]. Furthermore, post hoc analysis of the Look AHEAD randomized clinical trial demonstrated the effects of weight loss on CVD in overweight and obese T2DM with high risk for CVD [50]. Another previous study also reported that modest weight loss of 5–10% were associated with significant improvements in CVD risk factors at 1 year [51]. Thus, more than 5% loss of total body weight is recommended for overweight or obese people with T2DM in “Standards of Medical Care in Diabetes” of the American Diabetes Association as it can improve glycemic control, reduce the need for diabetes medications, and improve cardiovascular risk factors [52]. However, achieving this level of weight loss requires intense interventions, including energy restriction, regular physical activity, and frequent contact with health professionals [53]. In addition, 3% weight reduction is reported as the minimum requirement to improve health hazards at least in obese and overweight Japanese people [54]. Indeed, our previous studies showed body weight reduction of 2.8 ± 1.9% and 0.0 ± 2.8% relative to baseline in 16 weeks by dapagliflozin and linagliptin, respectively [25, 30]. Therefore, we set 3% loss in body weight as a component of the primary endpoint in the present study.

In addition to reduction in HbA1c and body weight loss, hypoglycemia is set as a component of primary endpoint. In the Action in Diabetes and Vascular Disease: Preterax and Diamicron Modified Release Controlled Evaluation (ADVANCE) study, severe hypoglycemia was clearly associated with increased risk of macrovascular events and death from cardiovascular cause [11]. It was also reported that hypoglycemia might cause CVD or death through abnormal cardiac arrhythmias [55], sympathoadrenal activation [56], inflammation [56], autonomic-failure [57], and impaired cardiac autoimmune function [58]. Recently, the International Hypoglycemia Study Group reported that blood glucose concentration of < 3.0 mmol/L (< 54 mg/dL) is considered to be clinically significant biochemical hypoglycemia and recommended to be included in reports of clinical trials of glucose-lowering drugs [59].

While we expect to compare the effects of dapagliflozin and sitagliptin on cardiovascular risk factors, such as glycemic control, body weight loss, and hypoglycemia, this study will have several limitations. First, as this is an open-label treatment and subjects can also know the detail of the medications used. Second, all patients will be Japanese and the duration of study is short. Therefore, additional long-term trials of larger sample size that preferably include subjects of different ethnicities, are desirable. Third, there are no patients who are treated with insulin in this study. We aimed to compare the effect of DPP4 inhibitor and SGLT2 inhibitor on the improvement of cardiovascular risks in T2DM with metformin or drug naive. This study may indicate the potential of each medication as a choice for first or second line treatment. However, the effect of add-on therapy to insulin remains to be studied.

In conclusion, the DIVERSITY-CVR study is planned to demonstrate the differential efficacy of dapagliflozin compared to sitagliptin on cardiovascular risk factors, such as glycemic control, body weight and hypoglycemia, in patients with T2DM. There are no studies that assessed such cardiovascular risk factors as a composite primary endpoint. Notably, the subjects of this studies are treated with only metformin or no glucose lowering agents before enrolment, thus the results may support strict treatment at the early stage of diabetes. The results of this study may have a large impact on the treatment of T2DM, taking into consideration the cardiovascular complications. The results of this study will become available in 2019.

## Additional file

**Additional file 1.** List of 62 medical institutions participating in the study.

## Abbreviations

CVD: cardiovascular diseases
DPP-4: dipeptidyl peptidase 4
FGM: flash glucose monitoring system
FMD: flow-mediated dilation
SGLT2: sodium glucose cotransporter 2
T2DM: type 2 diabetes mellitus
