# Supplementary material for: Rationale and design of study of dapagliflozin versus sitagliptin treatment efficacy on prevention of cardiovascular risk factors in type 2 diabetes patients: the DIVERSITY-CVR study
Source: Cardiovasc Diabetol. 2018 Jun 12;17:86. doi: 10.1186/s12933-018-0730-z (PMC5996551; doi:10.1186/s12933-018-0730-z)
Supplement: Supplementary file 1 — Additional file 1. List of 62 medical institutions participating in the study. [file 12933_2018_730_MOESM1_ESM.docx]

**Additional file 1**

Additional Supporting Information for the online version of this article:

**List of 62 medical institutions participating in the study: (listed in alphabetical order):**

- Amane Clinic
- Ayame Medical Clinic
- First Department of Internal Medicine, School of Medicine, University of Occupational and Environmental Health
- Fuchigami Clinic
- Fukuoka University Hospital
- Hasegawa Naika
- Heiwa Hospital
- Ikeda Clinic
- Ikegami general hospital
- Inokuchi Internal Medicine Clinic
- Isuzu Hospital
- Iwasaki Clinic
- Japanese Red Cross Kyoto Daiichi Hospital
- Japanese Red Cross Medical Center
- JCHO Tokyo Kamata Medical Center
- Johnin Ueda Clinic
- Juntendo Tokyo Koto Geriatric Medical Center
- Kajimoto Clinic
- Kawasaki Municipal Hospital
- Kawasaki Naika Hospital
- Kawasaki Rinko Hospital
- Keihin Hospital
- Kishida Clinic
- Kita Shinagawa 3rd. Hospital
- Kosugi Medical Clinic
- Kotani Diabetes Clinic
- Kumagaya Surgical Hospital
- Kumanomae Nishimura Clinic
- Makita General Hospital
- Manome Clinic
- Mashiba Clinic
- Matsubara Clinic
- Matsuda Clinic
- Mimiharatakasago Clinic
- Musashino Family Clinic
- Nagoya University, Graduate School of Medicine, Endocrinology and Diabetes
- Nakamichi Clinic
- Nakata Clinic
- Nishi Yokohama International Hospital
- Nissay Hospital
- NTT West Osaka Hospital
- Oomorinaka Shinryojyo
- Saiseikai Kanagawaken Hospital
- Saiseikai Yokohamashi Tobu Hospital
- Saitama Medical University Hospital
- Sakado Naika Iin
- Sapporo Diabetes Thyroid Clinic
- Sato Hospital
- Sawaki Internal Medicine and Diabetes Clinic
- Sekine Clinic
- Shimizu Clinic
- Shonan Hospital
- Taniguchi Clinic
- Toho University School of Medicine
- Tokyo Metropolitan Cancer and Infectious Diseases Center Komagome Hospital
- Tottori University Hospital
- Tsujinaka Clinic
- Tsukuba Diabetes Center Kawai Clinic
- Wada Clinic
- Wakaba Eye Hospital
- Yamamoto Clinic
- Yayoi Medical Clinic
